# Crystal Phase Ionic Liquids for Energy Applications: Heat Capacity Prediction via a Hybrid Group Contribution Approach

**DOI:** 10.3390/molecules29092130

**Published:** 2024-05-03

**Authors:** Moh’d Basel Shahin, Shehzad Liaqat, Paul Nancarrow, Sarah J. McCormack

**Affiliations:** 1 Department of Chemical and Biological Engineering, American University of Sharjah, Sharjah P.O. Box 26666, United Arab Emirates; b00085301@aus.edu (M.B.S.); sliaqat@aus.edu (S.L.); 2 Department of Civil, Structural and Environmental Engineering, Trinity College Dublin, D02 PN40 Dublin, Ireland; mccorms1@tcd.ie

**Keywords:** ionic liquids, heat capacity, crystal phase, group contribution, predictive model

## Abstract

In the selection and design of ionic liquids (ILs) for various applications, including heat transfer fluids, thermal energy storage materials, fuel cells, and solvents for chemical processes, heat capacity is a key thermodynamic property. While several attempts have been made to develop predictive models for the estimation of the heat capacity of ILs in their liquid phase, none so far have been reported for the ILs’ solid crystal phase. This is particularly important for applications where ILs will be used for thermal energy storage in the solid phase. For the first time, a model has been developed and used for the prediction of crystal phase heat capacity based on extending and modifying a previously developed hybrid group contribution model (GCM) for liquid phase heat capacity. A comprehensive database of over 5000 data points with 71 unique crystal phase ILs, comprising 42 different cations and 23 different anions, was used for parameterization and testing. This hybrid model takes into account the effect of the anion core, cation core, and subgroups within cations and anions, in addition to the derived indirect parameters that reflect the effects of branching and distribution around the core of the IL. According to the results, the developed GCM can reliably predict the crystal phase heat capacity with a mean absolute percentage error of 6.78%. This study aims to fill this current gap in the literature and to enable the design of ILs for thermal energy storage and other solid phase applications.

## 1. Introduction

Ionic liquids (ILs) are esoteric materials that have gained interest in recent decades for a variety of uses in chemistry and chemical engineering [1]. ILs are a type of salt that melts at ambient temperatures, generally considered to be temperatures lower than 373.15 K [2]. They are solely composed of cations and anions that can be combined in various arrangements [3]. Each synthesized cation–anion combination yields a unique IL that possesses distinct properties that may be useful for different applications [4]. Thus, ILs are often referred to as “designer” liquids due to their ability to have their physical and chemical properties specifically tuned for different purposes [1,4]. The most prominent features of ILs that make them a viable replacement to volatile organic compounds (VOCs) are their low volatility, thermal stability, non-flammability, and recyclability [5,6]. These versatile substances have the potential to be utilized in various industries and applications, including batteries, fuel cells, supercapacitors, lubricants and surfactants, food processes, drug delivery, and even carbon capture and water treatment processes [7,8,9,10,11,12,13].

It is important to study the thermophysical and transport properties of ILs in order to better comprehend their behavior and optimize their selection and design for various applications. Properties such as density, heat of fusion, thermal conductivity, melting point, and heat capacity play a vital role in determining the suitability and efficiency of an IL in different uses. Heat capacity is an essential thermodynamic property of an IL that is greatly affected by the existence of intermolecular forces between its ions, particularly hydrogen bonds [14]. This is true for most other thermophysical properties where the extent of hydrogen bonding in the ILs affects their physical and chemical characteristics [15,16]. The heat capacity has been shown to be highly dependent on the type of ions that make up the IL [17]. The variation in heat capacity can provide an understanding of the structure of the IL as well as its phase transition [18]. Moreover, it can help explain how the temperature impacts phase and reaction equilibria [17]. Applications where IL properties in the solid phase are important, such as phase change materials (PCMs) for thermal energy storage, require knowledge of the solid phase heat capacity for their optimal selection and design.

According to the literature, there are over a million potential pure ILs that can be synthesized and tested [19,20,21], along with IL ternary mixtures that are theoretically feasible [20,22,23,24]. Given this vast number, it is impractical to experimentally explore the viability of every possible IL due to time and other constraints [25]. A solution is to develop a predictive model that allows for the accurate estimation of IL properties without the need for experimental synthesis. There are different types of predictive models for IL property prediction that fall into three main categories, namely empirical models, theoretical models, and semi-empirical models. Theoretical predictive models utilize molecular models for estimations of properties such as quantum chemistry and thermodynamic relations in order to delve into the fundamental principles of compounds [26]. Empirical predictive models, on the other hand, rely on functional relationships that can capture specific datasets [26]. Finally, semi-empirical predictive models utilize a model equation in conjunction with the regressed structural parameters of the molecule for property estimation [26]. They are the most commonly utilized models when the prediction relies on an extensive number of parameters [27]. The two main semi-empirical approaches used for the prediction of ILs’ thermophysical properties are Quantitative Structure–Property Relationship (QSPR) models [28] and group contribution models (GCMs) [29]. The QSPR model is a predictive method that relies on “descriptors” which are chemical or physical properties that are derived from quantum calculations [27,30]. On the other hand, the GCM is a technique that utilizes the occurrence of the functional groups and particular groups of atoms within the molecular structure of the chemical species in order to predict its properties [31]. The GCM approach usually assumes that the contributions of the structural groups in the species are additive. The additive approach has been demonstrated to be effective in predicting certain thermophysical properties, such as density and heat capacity [27], but it proves less adequate for others, such as melting point, as these properties cannot be accurately described by the additive contributions of functional groups in the molecular structure of the species [27,30].

In comparison to QSPR models, GCMs do not require computationally intensive quantum calculations for prediction because the parameters that are used for their prediction can be easily extracted from the molecular structure of the IL. GCM parameters also possess a straightforward and easily comprehensible physical interpretation, unlike the intricate descriptors used in QSPR models. The development of GCMs for IL property estimation was previously constrained by the range of functional group contribution parameters due to the limited availability of characterized ILs. Both the quantity of ILs and the diversity of functional groups included in the databases utilized for these GCMs were limited. Nevertheless, the significant increase in published IL data in recent years advocates for the further research efforts aimed at reevaluating GCMs for IL property prediction and potentially improving the GCM approach. Furthermore, the GCM approach takes into consideration the influence of the cation–anion structure [17], individual functional groups, and the length of the alkyl chain [32] within the IL which proves to be highly suitable for heat capacity prediction, as heat capacities are properties that are greatly affected by the nature of the cation and anion and the overall structure of the IL [14].

Various studies have developed GCMs for estimating the heat capacity of ILs in the liquid phase [17,31,32,33,34,35,36,37,38,39,40,41,42,43,44]; however, to the best of the authors’ knowledge, there are currently no GCMs specifically developed for the heat capacities of ILs in the solid phase. Hence, in this study, a GCM was developed for the heat capacity of solid crystal phase ILs to address this existing gap in the literature and to encourage the potential development of future thermal energy storage applications for solid phase ILs. The database compiled for this work consists of over 5000 data points, featuring 71 unique ILs, with 42 unique cations and 23 unique anions. Furthermore, to improve upon the traditional GCM method, a hybrid GCM-QSPR approach is used, which combines functional group parameters with additional structural parameters that describe more complex aspects of the structure–property relationships without the need for computationally expensive quantum calculations. This approach is essential, since it is anticipated that the model will, in the future, be incorporated into an IL design framework capable of screening a very large search space of around 10^6^ hypothetical IL structures within reasonable time frames.

The GCM aspect of this model builds upon and extends the Lazzus model [45]. Further improvements were achieved by introducing a range of indirect structural parameters, such as the number of chains, the size parameter, the number of rings, and the total number of carbons within the cation core, in an approach similar to that previously developed for the estimation of the melting points and liquid phase heat capacities of pure ILs [31,46]. The model’s performance was assessed, and an analysis was conducted to investigate the influence of the structural parameters on crystal phase heat capacity prediction.

## 2. Results

### 2.1. Database Development and Data Analysis

The IL database used in this study was sourced from NIST ILThermo [46], where 5007 data points were extracted from 38 references from within the literature [47,48,49,50,51,52,53,54,55,56,57,58,59,60,61,62,63,64,65,66,67,68,69,70,71,72,73,74,75,76,77,78,79,80,81,82,83,84] that have reported experimental measurements of the heat capacities of crystal phase ILs. Within the 5007 data points, there are 71 unique ILs comprising 42 total cations and 23 total anions. The heat capacity values in the database range from 17.89 J∙mol^−1^∙K^−1^ to 1438 J∙mol^−1^∙K^−1^ which were measured at atmospheric pressure and temperatures that range from 12.45 K to 403 K. Prior to utilizing the database, data cleaning was conducted by carefully eliminating duplicate data points in order to avoid redundancy and ensure the reliability of the model. To leverage the database for the development of the GCM, the programming language Python was utilized. The pandas Python package (version 3.10) [85] was used to import the database as a DataFrame. Ultimately, the comprehensive analysis and visualization of the data were performed using the seaborn (version 0.12.0) [86] and matplotlib (version 3.7.0) [87] Python packages.

**Figure 1:**
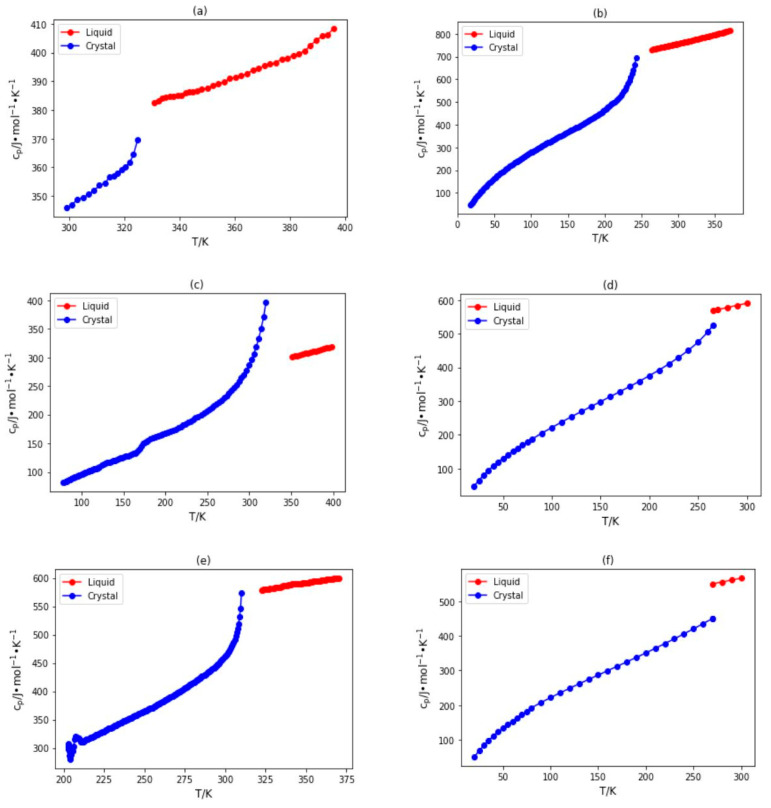
Plots of heat capacity versus temperature for ILs in the crystal and liquid phases, including (**a**) 1-pentylpyridinium hexafluorophosphate [56], (**b**) 1-decyl-3-methylimidazolium bis(trifluoromethylsulfonyl)amide [51], (**c**) 1-propylpyridinium bromide [59], (**d**) N-butyl-N-methylpyrrolidinium bis(trifluoromethylsulfonyl)amide [86], (**e**) 1-hexylquinolinium bis(trifluoromethylsulfonyl)amide [53], and (**f**) 1-butyl-3-methylimidazolium bis(trifluoromethylsulfonyl)amide [86].

Figure 1 shows the IL heat capacity in the crystal phase and the liquid phase for several different ILs. It is clear that there is a discontinuity between the phases in terms of heat capacity. Furthermore, the temperature dependence of heat capacity is somewhat different in each phase. This gives a strong indication that the existing liquid phase heat capacity models cannot be used directly for the prediction of IL heat capacity in the crystal phase. Therefore, there is a strong motivation to develop a new model for IL heat capacity in the crystal phase.

**Figure 2:**
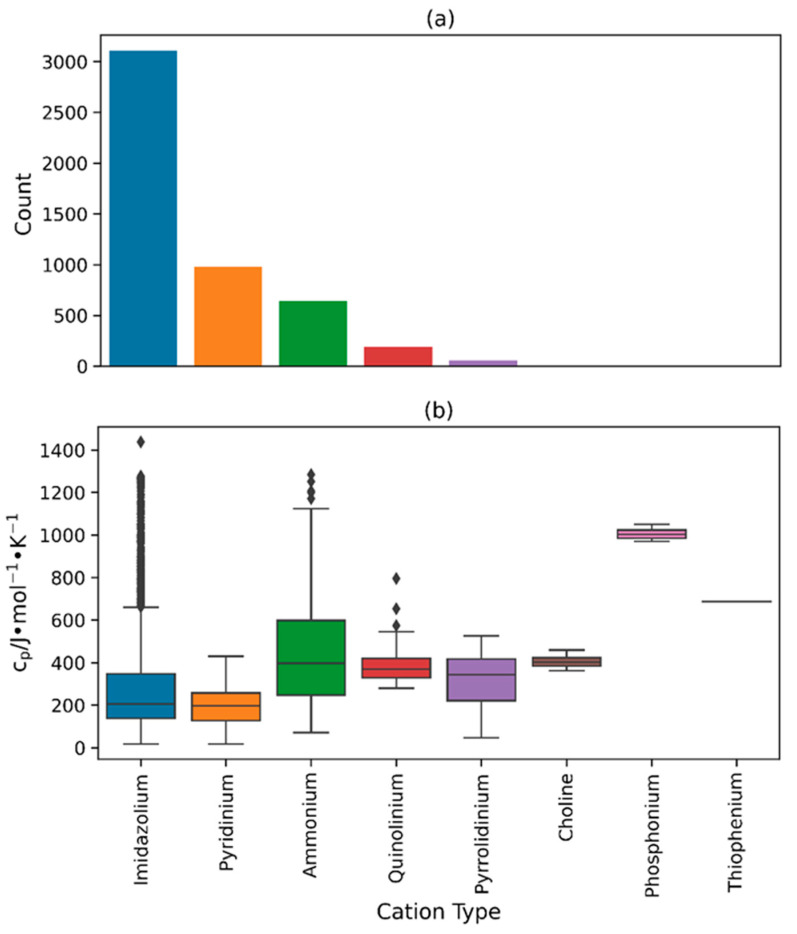
(**a**) Bar plot illustrating the total count of each cation type within the database, and (**b**) box and whisper plot illustrating the spread of crystal phase heat capacity values across each cation type.

**Figure 3:**
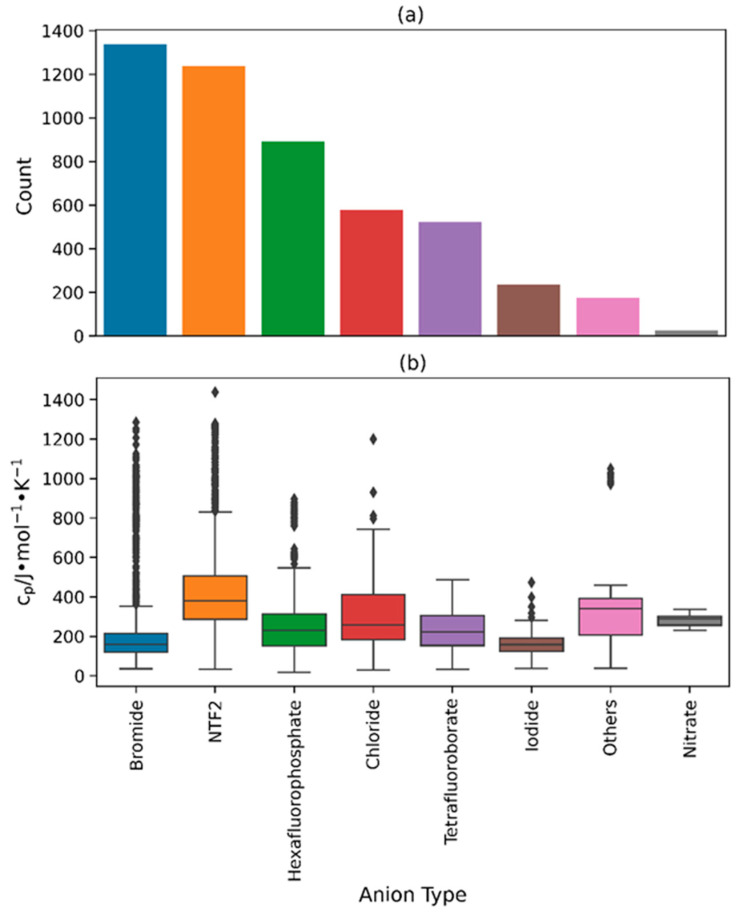
(**a**) Bar plot illustrating the total number of each anion type in the database, and (**b**) box and whisper plot illustrating the spread of crystal phase heat capacity values across each anion type.

Figure 2a and Figure 3a depict the quantity of different cation and anion types within the heat capacity database. The most common cation type in the database is imidazolium (3107 data points) while the most common anion type is bromide (1337 data points). The order of the cation types that follow, as shown in the bar plot, is pyridinium (983 data points), ammonium (647 data points), quinolinium (194 data points), and pyrrolidinium (57 data points), with relatively lower occurrences of other cations like choline (11 data points), phosphonium (7 data points), and thiophenium (1 data point). Similarly, the order of anion types follows the sequence of bis((trifluoromethyl)sulfonyl)imide (NTF2) (1239 data points), hexafluorophosphate (893 data points), chloride (578 data points), tetrafluoroborate (523 data points), and iodide (236 data points), with relatively smaller quantities of nitrate (26 data points) and other less prevalent anion types that fall under the “Others” category (175 data points). This is consistent with the demand and relevance of the more frequently occurring types of ILs for research and industrial applications, including imidazolium-, NTF2-, and bromide-based ILs [3,88,89,90]. Table 1 lists the anion types from the “Others” category along with their number of data points and references.

The box and whisker plots in Figure 2b and Figure 3b demonstrate the distribution of heat capacity values for every type of cation and anion found in the database. In terms of cation types, phosphonium-based ILs exhibited the highest average heat capacity compared to all other cation types, with an approximate value of 1000 J∙mol^−1^∙K^−1^. Conversely, pyridinium- and imidazolium-based ILs possessed the lowest average heat capacity, roughly 200 J∙mol^−1^∙K^−1^. This significant difference in the average heat capacity values can be attributed to the difference in average molecular weights of these IL types. The average molecular weight of phosphonium-based ILs in the database is 704.45 g∙mol^−1^ which is much larger than the average molecular weights of pyridinium- and imidazolium-based ILs which are equal to 227.22 g∙mol^−1^ and 331.46 g∙mol^−1^, respectively. It is also possible that the variations in temperature ranges among these IL types caused this disparity (283.15 K to 328.15 K for phosphonium, 12.45 K to 363.15 K for imidazolium, and 15.85 K to 371.50 K for pyridinium) since the heat capacity tends to increase with an increase in the chain length and temperature [32]. It should be noted that the database primarily comprises imidazolium-based ILs, while phosphonium- and pyridinium-based ILs represent a relatively smaller proportion. Furthermore, imidazolium-based ILs possess the widest range of heat capacity values, with a range of 18.11 J∙mol−1∙K^−1^ to 1438.0 J∙mol^−1^∙K^−1^. As for the anion types, NTF2-based ILs stand out with the highest median heat capacity (approximately 400.0 J∙mol^−1^∙K^−1^) as well as the widest range of heat capacity (32.90 J∙mol^−1^∙K^−1^ to 1438.0 J∙mol^−1^∙K^−1^) in the database. On the other hand, bromide-based ILs, which are the most common in terms of anions, exhibit the lowest median heat capacity of approximately 185.0 J∙mol^−1^∙K^−1^. With the exception of iodide- and nitrate-based ILs, the majority of anion types generally possess a wide range of heat capacity values.

**Figure 4:**
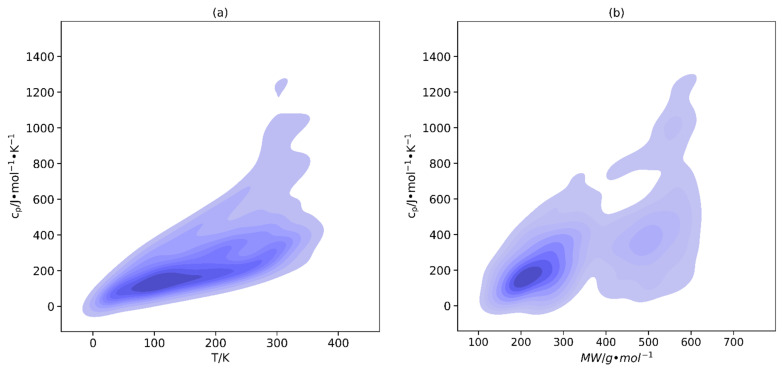
Kernel density estimate plots depicting the effects of (**a**) temperature and (**b**) molecular weight on the heat capacity of crystal phase ILs.

Figure 4 shows the effect of temperature and molecular weight on the heat capacity of ILs. According to the plots, a trend can be observed indicating that as the temperature and molecular weight of an IL rises, there is a corresponding increase in its heat capacity. This agrees with what has been already demonstrated in the literature [17,91]. Therefore, it can be deduced that the heat capacity of an IL is significantly influenced by both its molecular weight and temperature. As a result, these factors must be taken into consideration when selecting an IL for an application that requires a specific range of heat capacity, and also in the development of any predictive model.

**Figure 5:**
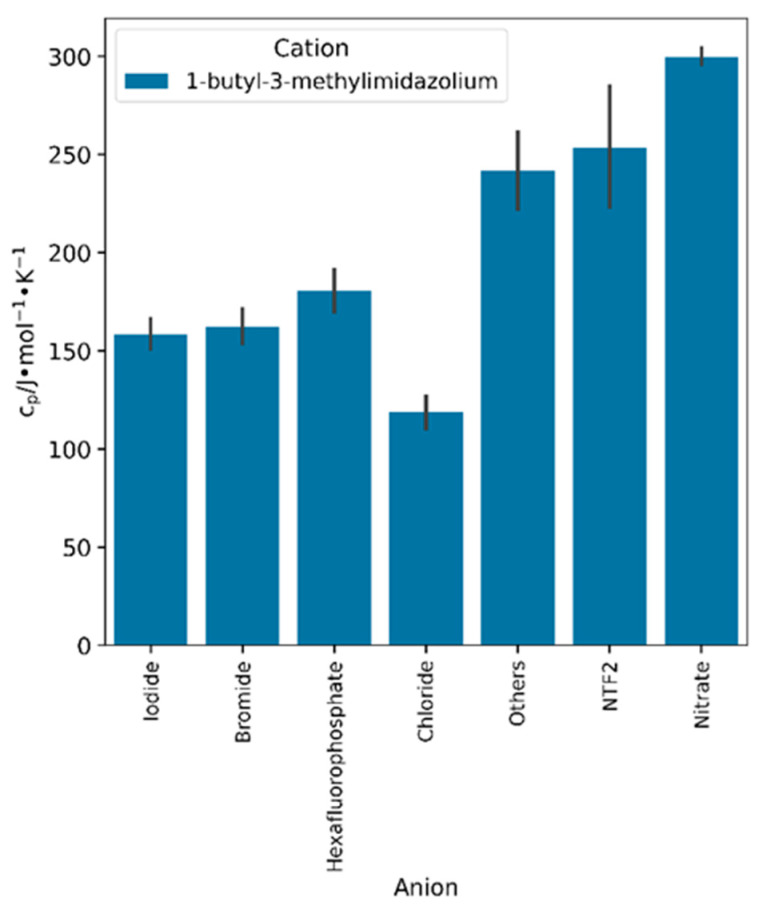
Effect of anion type on the heat capacity of 1-butyl-3-methylimidazolium-based ILs.

The influence of the anion and its molecular weight on the heat capacity of the IL is known to be more significant compared to the cation [32,92]. In order to assess the impact of the anion core on the heat capacity of crystal phase ILs, the heat capacity of 1-butyl-3-methylimidazolium-based ILs is compared with different anion types in Figure 5. The trend observed from the plot in ascending order is nitrate > NTF2 > others > hexafluorophosphate > bromide > iodide > chloride. Apart from iodide and nitrate, the findings are consistent with the growing linear relationship between the heat capacity and the molecular weight of the anion. Moreover, previous studies have reported similar trends [17,48,93,94]. The heat capacities of iodide and nitrate were considerably affected by the average temperature of the data points in comparison to the effect of their molecular weights. The reason for the high heat capacity of nitrate is its high average temperature (291.3 K) compared to the other anion types. Similarly, iodide has a lower average heat capacity than bromide despite having a higher molecular weight because it has a lower average temperature (163.7 K for iodide and 176.0 K for bromide).

### 2.2. Model Development

To estimate the heat capacity of ILs in the crystal phase, a hybrid GCM approach similar to that previously applied for IL liquid phase predictions was employed [31]. This GCM accounts for the influence of cation and anion cores, as well as subgroups within cations and anions. In order to accommodate the effects of branching and dispersion around the core, the indirect parameters that have been derived were integrated within the model.

Initially introduced for predicting the melting points of pure ILs [31], these indirect parameters were intended to address the limitations encountered in earlier GCMs and improve the predictive capabilities of the model. The heat capacity of substances is influenced by various parameters, including the ionic structure, functional groups, size, and intermolecular and intramolecular forces, all of which play crucial roles in determining their physical and chemical properties as well as their stability [14,15,16,17,44,76]. The heat capacity is also known to be strongly influenced by factors such as molecular weight, alkyl chain length, and the types of cation and anion cores [32]. Therefore, it is expected that incorporation of these parameters will improve the accuracy of the GCM as they more accurately reflect the effect of intermolecular forces and inner interactions, as well as the specific structural effects that are often overlooked in the group contribution method. The description of each derived parameter included in the developed GCM is detailed in the Supporting Information (Table S1).

The GCM developed for the prediction of the crystal phase heat capacity incorporates a broad range of anion and cation cores. All the parameters utilized in the GCM are listed in Supplementary Materials S1 (Table S2). Moreover, Supplementary Materials S1 and Supplementary Materials S2 contain the group contribution values for each parameter used in the GCM as well as an example of how these parameters were used to compute and validate the predicted heat capacity values (provided in an Excel file). 

The influence of temperature has been taken into account using a second-order temperature dependence relationship was chosen. This relationship has been proven to accurately depict how temperature affects the heat capacity of ILs [17]. Additionally, this approach allows the crystal phase heat capacity of ILs to be estimated across a wide temperature range, spanning from 12.45 K to 403.0 K. The relationship is shown in the Equations (1)–(4) below:

(1) $$C_{P}=R[A+B(\frac{T}{100})+C{\left(\frac{T}{100}\right)}^{2}]$$

(2) $$A=\Sigma (a_{i}\times n_{i})$$

(3) $$B=\Sigma (b_{i}\times n_{i})$$

(4) $$C=\Sigma (c_{i}\times n_{i})$$

where *a*_i_, *b*_i_ and *c*_i_ are the contribution parameters of the *i*th group, *n*_i_ is the frequency of the occurrence of the *i*th group, and *R* is the universal gas constant which is equal to 8.314 J∙mol^−1^∙K^−1^. Supplementary Materials S1 contains a detailed explanation of the model, a complete table of values for the model’s parameters, and an example of how to use the model.

In order to prepare the datasets for model fitting, they were first split into an 80:20 training set-to-testing set ratio. A number of metrics were used to assess the model’s performance, including the mean absolute error (MAE), root mean square error (RMSE) [95], mean square error (MSE) [96], mean absolute percentage error (MAPE) [97], and coefficient of determination (*R*^2^) [98] for the testing, training, and overall data. Nevertheless, the MAE was the only metric chosen as the objective function to be minimized over the training set in order to reduce the impact of outliers. Using the following error formulae, the model’s effectiveness was evaluated [95,96,97]:

(5) $$MAE=\frac{1}{N}\times \Sigma \left|C_{P,pred}-C_{P,exp}\right|$$

(6) $$RMSE=\sqrt{\frac{1}{N}\times \Sigma {\left(C_{P,pred}-C_{P,exp}\right)}^{2}}$$

(7) $$MSE=\frac{1}{N}\times \Sigma {\left(C_{P,pred}-C_{P,exp}\right)}^{2}$$

(8) $$MAPE=(\frac{1}{N}\times \Sigma \frac{\left|C_{P,pred}-C_{P,exp}\right|}{\;C_{P,exp}})\times 100\%$$

where *C*_(*P,pred*)_ is the predicted value of heat capacity, *C*_(*P,exp*)_ is the experimental value of heat capacity, and *N* is the number of points in the dataset.

The scikit-learn Python package [99] was used for multiple linear regression. Moreover, scikit-learn was utilized because its algorithm uses gradient descent which is specifically designed to converge towards a global minimum, ensuring the development of an optimal solution for the model [100]. The main aim of the algorithm is to minimize the cost function. The cost function of the heat capacity linear regression model is given by:

(9) $$Cost=\frac{\sum {\left(C_{P,pred}-C_{P,exp}\right)}^{2}}{2}$$

The gradient descent algorithm minimizes the cost function by continuously iterating and updating the weights of the model until a terminating condition is satisfied [100]. The gradient descent is guaranteed to converge to a global minimum, instead of a local minimum, in the function space in cases where the cost functions are convex, as demonstrated in the cost equation of the linear regression model [100].

## 3. Discussion

The performance metrics, MAPE and *R*^2^, were used to evaluate the developed GCM. As shown in Table 2, the resulting MAPE for the overall database was 6.78% and the *R*^2^ was 0.974. These results indicate that the GCM can predict the crystal phase heat capacity with reasonable accuracy. Moreover, it is important to note that the error of these MAPE and *R*^2^ values must be greater than the error of the experimental measurements for the heat capacity of the ILs in the database. This is because the uncertainty of the experimental measurements is added to the uncertainty that exists between the actual and predicted values of heat capacity. 

**Figure 6:**
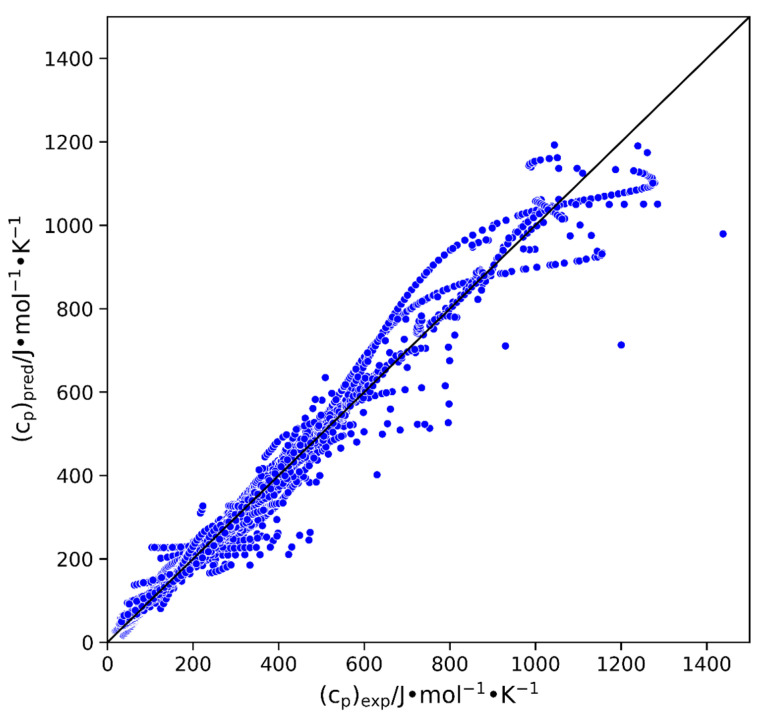
Parity plot of predicted heat capacity versus experimental heat capacity for the full dataset (~5000 points).

A parity plot of the developed GCM’s predicted heat capacity vs. its experimental heat capacity is shown in Figure 6. The black 45-degree line indicates where the data should lie when the model perfectly predicts the experimental data. The plot illustrates that most of the over 5000 data points are evenly distributed around the 45° line. However, there are a few anomalies, indicating instances where the model struggled to accurately predict the heat capacities of the crystal phase.

**Figure 7:**
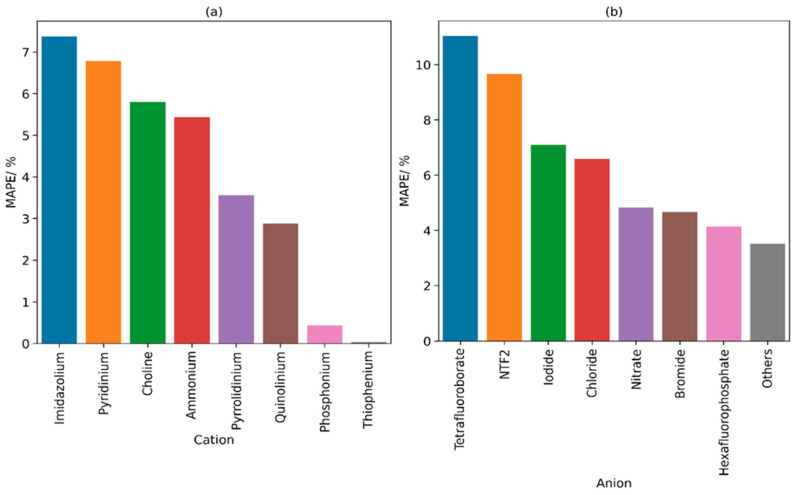
MAPE for ILs according to (**a**) cation and (**b**) anion core for the developed GCM when applied on the entire database.

Figure 7 illustrates the calculated MAPE for the model when tested against ILs with different cation and anion core types. In general, cation and anion groups with a higher abundance of data points tend to exhibit lower average errors. This can be attributed to the reduced uncertainty in the training set which prevents the model from overfitting. Similarly, groups with a limited number of data points tend to also show lower errors, primarily due to their representation of a smaller subset of ILs which makes the regression more simplified. Groups such as bromide, chloride, hexafluorophosphate, and ammonium have relatively smaller MAPE values because of their larger number of data points. According to the figure, the highest error is observed in imidazolium and tetrafluoroborate ILs while the lowest error is observed in thiophenium ILs and the “Others” category.

**Figure 8:**
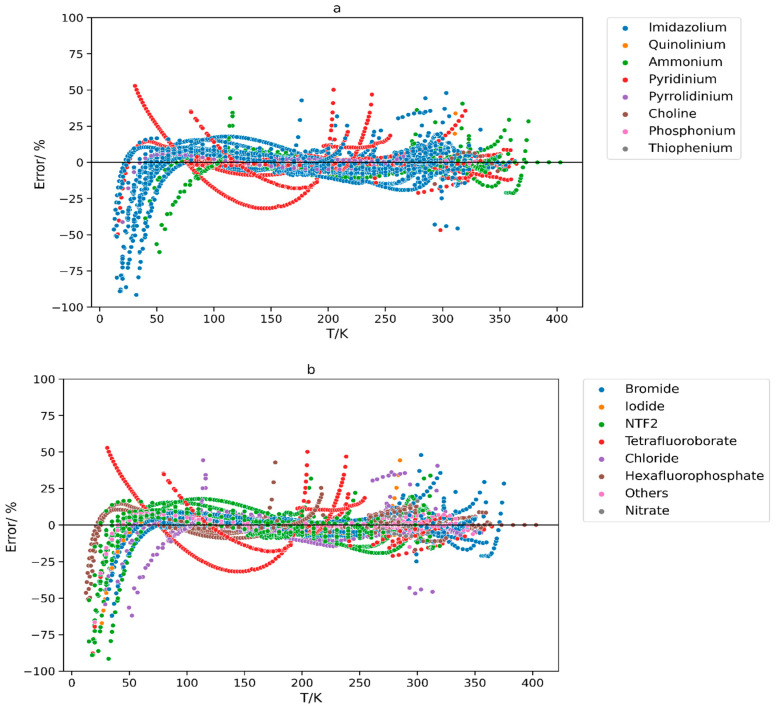
Residual plots of percentage error versus temperature for different (**a**) cation types, and (**b**) anion types when the hybrid GCM was applied.

The plots in Figure 8 illustrate the error percentages of different cation and anion groups at different temperatures. Residual plots serve as a valuable tool to evaluate the performance of GCMs in relation to their errors. The desired result is represented by points that are uniformly distributed around the plots’ x-axes. The plots showcase a relatively balanced distribution of data points along the x-axis, with some subtle patterns within the dataset. At temperatures below 100 K, the data points were distributed more towards the negative side, while in the temperature range of 100 K to 400 K, the points exhibited a relatively balanced distribution around the x-axis, with a few noticeable outliers. 

In both plots, a distinct outlier pattern is shown (red-colored points) which comes from the ILs pyridinium tetrafluoroborate [55] and 1-butylpyridinium tetrafluoroborate63. It is unclear whether the error originates from the model, or the data points themselves because the data for each of these two ILs were extracted from a single source. To enhance the credibility of the existing data and improve the accuracy of the model, further experimental measurements of these types of crystal phase ILs are required.

**Figure 9:**
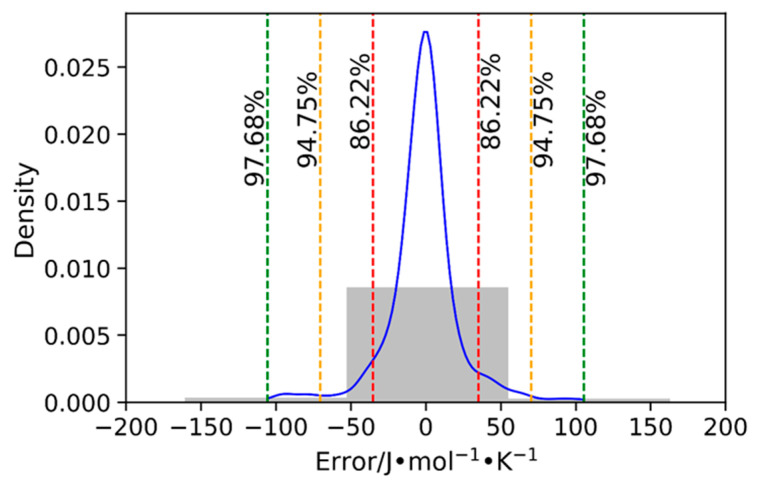
Density plot showing the error distribution for the hybrid GCM when applied on the entire database.

Figure 9 shows the distribution of error for the developed GCM when applied on the entire database. The histogram clearly indicates that most of the errors lie between −50 and 50 J∙mol−1∙K−1. Furthermore, the curve illustrates that a substantial portion, specifically 86.22%, of the entire database exhibits errors falling within the narrower range of −30 to 30 J∙mol−1∙K−1and the majority of the data points (94.75%) exhibit errors within the range of −70 to 70 J.mol^−1^K^−1^. This analysis indicates that the developed GCM exhibits an adequate level of accuracy and reliability in predicting the crystal phase heat capacities of ILs.

**Figure 10:**
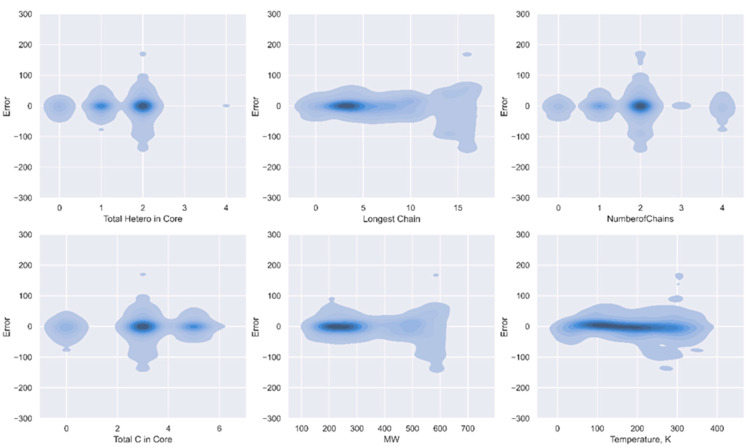
Kernel density estimate plots illustrating the distribution of residual errors for various parameters in the hybrid GCM.

The distribution of residual errors for different parameters in the developed GCM when applied on the whole database is shown in Figure 10. This type of plot can be used to analyze the performance of the model against the various structural characteristics of ILs that were used in constructing the hybrid GCM. The plots indicate that, for all parameters, the darker regions predominantly lie near or around zero (x-axis), suggesting that the model is performing well for most of the data. However, for the Longest Chain and MW parameters, there is a tendency of the GCM to underestimate the values as the MW increases (above 500 g/mol) and the longest chain extends (above 12). Nevertheless, it is evident that the developed GCM effectively captures the effects of these structural characteristics, indicating their positive influence of these structural parameters on the GCM’s performance.

## 4. Materials and Methods

An IL database of ILs’ crystal phase heat capacity as a function of temperature was compiled using the NIST ILThermo [46] as the main source, consisting of 5007 data points extracted from 38 references in the literature [47,48,49,50,51,52,53,54,55,56,57,58,59,60,61,62,63,64,65,66,67,68,69,70,71,72,73,74,75,76,77,78,79,80,81,82,83,84]. Data cleaning was conducted by carefully eliminating duplicate data points in order to avoid redundancy and ensure the reliability of the model. The pandas Python package (version 3.10) [85] was used to import the database as a DataFrame, while the comprehensive analysis and visualization of the data were performed using the seaborn (version 0.12.0) [86] and matplotlib (version 3.7.0) [87] Python packages. Multiple linear regression was performed using the scikit-learn (version 1.1.3) Python package [99]. It was selected because its algorithm uses gradient descent which is designed for convergence towards a global minimum [100]. All the parameters utilized in the GCM are listed in Supplementary Materials S1 (Table S2). Moreover, Supplementary Materials S1 and Supplementary Materials S2 contain the group contribution values for each parameter used in the GCM as well as an example of how these parameters were used to compute and validate the predicted heat capacity values (provided in an Excel file).

## 5. Conclusions

Given that an almost limitless number of ILs can be possibly synthesized and investigated, the utilization of reliable predictive models to estimate their physical and chemical becomes crucial to enable their optimal selection for various industrial applications. In this work, the group contribution modeling approach was applied to an entire database including over 5000 data points to predict the crystal phase heat capacity of ILs. The employed hybrid GCM involved a combination of conventional functional group parameters and easily derived indirect structural parameters. The assessment of the model’s performance involved analyzing its MAPE and *R*^2^ values, which were determined to be 6.78% and 0.974, respectively. It can be concluded that the developed hybrid GCM can be utilized to predict the heat capacity of crystal phase ILs to a reliable degree of accuracy as well as maintain a broad range of applicability. The findings indicate that the proposed hybrid GCM exhibits a reliable and accurate prediction capability for the heat capacity of crystal phase ILs, while also ensuring a wide range of applicability. It should be noted, however, that certain cases exhibit notable errors, which can be attributed to the uncertainties of the experimental measurements in the database. Furthermore, as the availability of IL experimental data increases, the model can be further refined to enhance its ability to predict the crystal phase heat capacity for a broad range of ILs.

## Figures and Tables

**Table 1:** List of anion types under the “Others” category.

Anion Type	Data Points	Reference
1,1,2,2-tetrafluoroethanesulfonate	20	[70]
2-methoxy-2-oxoacetate	7	[78]
4-methylbenzenesulfonate	18	[74,75]
Dimethylphosphate	5	[75]
Tosylate	50	[69,74]
Trifluoroacetate	30	[66]
Trifluoromethanesulfonate	38	[71]
Tris(pentafluoroethyl)trifluorophosphate	7	[79]

**Table 2:** Performance metrics of the developed GCM for the testing and overall datasets.

Metric	Test Set	Overall
Ratio of Database (%)	20	100
MAPE (%)	6.83	6.78
*R* ^2^	0.976	0.974

## Data Availability

The database of crystal phase ionic liquid heat capacities used in this study has been made available in the excel file (Supplementary Materials S2).

## Supplementary Materials

The following supporting information can be downloaded at: https://www.mdpi.com/article/10.3390/molecules29092130/s1, The description of the derived indirect parameters introduced into the hybrid GCM (Table S1), the functional group building blocks utilized (Table S2), the fitted group contribution and structural parameters for the hybrid GCM (Table S3), and a sample calculation (Table S4) has been provided in Supplementary Materials S1 as a word document. The dataset used and a sample calculation of heat capacity using the hybrid GCM has been provided in Supporting Information S2 as an excel sheet.
