# Supplementary material for: Crystal Phase Ionic Liquids for Energy Applications: Heat Capacity Prediction via a Hybrid Group Contribution Approach
Source: Molecules. 2024 May 3;29(9):2130. doi: 10.3390/molecules29092130 (PMC11085896; doi:10.3390/molecules29092130)
Supplement: Supplementary file 1 [file molecules-29-02130-s001.zip › Supporting Information 1-v2.BS.pdf]

Supporting Information 1

Crystal phase ionic liquids for energy applications: Heat capacity prediction via a hybrid group contribution approach

Moh'd Basel Shahin[1], Shehzad Liaqat[1], Paul Nancarrow[1,2] Sarah McCormack[2]

[1]*Department of Chemical & Biological Engineering, American University of Sharjah, PO Box 26666, Sharjah, United Arab Emirates*

[2]*Department of Civil, Structural and Environmental Engineering, Trinity College Dublin, Dublin 2, D02 PN40, Ireland*

**Corresponding author: Email: pnancarrow@aus.edu*

Table S1. List and description of the derived indirect parameters introduced into the GCM.

**Parameter**	**Definition**
$(SO_2)_2N$	An additional anion subgroup to consider its effect on the heat capacity.
MW	The IL's molecular weight which accounts for the effect of size on the heat capacity.
Normalized MW	The normalized molecular weight of the IL:
Total C in Core	The count of carbon atoms in the cation core which considers how the number of carbon atoms in the cation core affects the heat capacity.
Longest Chain	The carbon atom count in the longest chain of the IL which considers the influence on the heat capacity based on the hydrocarbon chain length.
Number of Chains	The number of chains in the IL which considers the impact of structural symmetry and weight distribution on the heat capacity.
Long Chain	The molecular weight of the longest chain which accounts for the change in heat capacity based on the variations in chain lengths.
Ln (long chain)	The natural logarithm of the molecular weight of the longest chain which incorporates the effect of both the positive and the negative values.
Ln (MW of Cation)	The natural logarithm of the molecular weight of the cation which accounts for how the cation's structure affects the heat capacity.

Size Parameter	The ratio of the molecular weight of the longest cation chain to the molecular weight of the entire cation which takes into account the impact of weight distribution on the heat capacity.
Cation/Anion	The ratio of the molecular weight of the cation to that of the anion which considers the impact of cation and anion size difference on the heat capacity.
All Other/LongChain	The ratio of all other groups attached to the cation core, excluding the longest chain, to the weight of the longest chain which reflects the impact of structural symmetry on the heat capacity.
AllOther	The weight of all additional groups connected to the cation core, excluding the longest chain.
Total Hetero in Core	The count of heteroatoms in the cation core, excluding carbon and hydrogen which captures the heat capacity variation associated with non-carbon and non-hydrogen heteroatoms in the cation core.
Total Cation Cores	The total number of cation cores which considers how the heat capacity changes with the number of cation cores.
Number of Rings	The number of rings in the IL which considers any variations in heat capacity based on the presence of aromatic rings.
Ring CH (Non-Core)	The number of CH rings outside the cation core which takes into account their contribution towards the heat capacity.

Table S2. Functional group building blocks utilized in the GCM.

	**Cation Cores**	**Cation Subgroups**	**Anion Subgroups**	**Added Indirect Parameters**
**GCM**	Imidazolium	–H	–CH_3.1	MW
	Pyridinium	–CH_3	–CH_2–.1	NormalizedMW
	Pyrrolidinium	–CH_2–	〉CH–	Total C in Core
	Piperidinium	–CH〈	〉C〈.1	Total Hetero in Core
	Ammonium	–OH	〉CO	TotalCationCores
	Phosphonium	–O–	–COO–	Number of Rings
	Thiophenium	〉C〈	–HCOO	Ring CH (Non-Core)
	Quinolinium	–COO	–OH.1	Longest Chain
	Azepanium	–CN	–O– [–O]	NumberofChains
		–NH_2	–CN.1	LongChain
		–SO_2–	–N– [〉N–]	lnlongchain
		Phenyl	–NO_2	AllOther/LongChain
		–Benzyl	–S–	AllOther

		–Vinyl	$–SO_2–.1$	Size Parameter
		$–CF_2–$	$–CF_3.1$	Cation/Anion
		$–CF_3$	$–CF_2–.1$	lnMWCation
			–F.1	$(SO_2)_2N$
			–Cl.1	
			–Br.1	
			–P	
			–B	
			–I	
			CH– (ring)	
			C– (ring)	
			〉CO (ring)	
			–O– (ring)	

Table S3. Group contribution parameters of each group for developed model.

Groups	a_i	b_i	c_i
Imidazolium	-1.22E+03	8.34E+02	-1.47E+02
Pyridinium	2.61E+03	-1.72E+03	2.90E+02
Pyrrolidinium	-3.22E+03	2.10E+03	-3.49E+02
Piperidinium	1.01E-03	-2.50E-06	0.00E+00
Ammonium	7.40E+00	2.64E+01	-1.87E+01
Phosphonium	-3.12E+02	3.74E+02	-9.80E+01
Thiophenium	-2.86E-01	-8.43E-01	-2.51E+00
quinolinium	4.77E+02	-3.15E+02	5.38E+01
Azepanium	4.20E-04	0.00E+00	0.00E+00
–H	1.19E+03	-7.75E+02	1.29E+02
–CH3	2.18E+03	-1.42E+03	2.23E+02
–CH2–	-1.13E+02	1.36E+02	-3.35E+01
–CH〈	2.98E-03	-2.79E-09	0.00E+00
–OH	2.95E+02	-7.86E+01	-2.16E+01
–O–	-5.46E-05	0.00E+00	0.00E+00
〉C〈	1.47E-04	0.00E+00	0.00E+00
–COO	-4.07E-05	0.00E+00	0.00E+00
–CN	-9.01E-01	-2.69E+00	-8.00E+00

–NH2	5.74E-05	0.00E+00	0.00E+00
–SO2–	3.28E-05	0.00E+00	0.00E+00
Phenyl	4.14E-06	0.00E+00	0.00E+00
–Benzyl	-1.63E+03	1.23E+03	-2.43E+02
–Vinyl	2.89E-06	0.00E+00	0.00E+00
–CF2–	8.73E-05	0.00E+00	0.00E+00
–CF3	-7.61E-05	0.00E+00	0.00E+00
–CH3.1	-6.15E+02	7.32E+02	-1.87E+02
–CH2–.1	-2.91E-05	0.00E+00	0.00E+00
〉CH–	-9.95E-06	0.00E+00	0.00E+00
〉C〈.1	-5.36E-07	0.00E+00	0.00E+00
〉CO	-9.30E-06	0.00E+00	0.00E+00
–COO–	6.12E+01	-1.39E+02	4.17E+01
–HCOO	8.05E-05	0.00E+00	0.00E+00
–OH.1	8.04E-05	0.00E+00	0.00E+00
–O– [–O]	8.58E+01	-1.75E+02	5.24E+01
–CN.1	-6.24E-05	0.00E+00	0.00E+00
–N– [〉N–]	-2.44E-05	0.00E+00	0.00E+00
–NO2	-4.71E+02	4.50E+02	-1.07E+02
–S–	-5.64E-05	0.00E+00	0.00E+00
–SO2–.1	-3.33E+02	3.63E+02	-8.90E+01
–CF3.1	-7.33E+02	7.44E+02	-1.77E+02
–CF2–.1	-5.21E+02	5.32E+02	-1.26E+02
–F.1	-1.51E+02	1.40E+02	-3.17E+01
–Cl.1	-1.38E+02	1.09E+02	-2.64E+01
–Br.1	-4.05E+02	3.27E+02	-7.11E+01
–P	-4.12E+01	6.17E+01	-1.66E+01
–B	1.23E+02	-1.48E+02	3.65E+01
–I	-7.88E+02	7.19E+02	-1.63E+02
CH– (ring)	-6.39E+01	4.65E+01	-8.68E+00
C– (ring)	-3.19E+01	2.32E+01	-4.34E+00
〉CO (ring)	-1.11E-05	0.00E+00	0.00E+00
–O– (ring)	-2.29E-06	0.00E+00	0.00E+00
MW	2.27E+07	-2.05E+00	1.11E+00
NormalizedMW	-3.32E+09	-1.38E+03	2.67E+02
Total C in Core	-1.10E+03	7.37E+02	-1.27E+02
Total Hetero in Core	2.81E+03	-1.84E+03	3.11E+02
TotalCationCores	-1.66E+03	1.30E+03	-2.71E+02
Nitro	-7.64E-03	2.78E-05	0.00E+00
Number of Rings	-2.50E+03	1.81E+03	-3.44E+02
Ring CH (Non-Core)	1.91E+03	-1.26E+03	2.15E+02

Longest Chain	-4.74E+01	-4.15E+00	7.28E+00
NumberofChains	-7.89E+02	5.27E+02	-7.50E+01
LongChain	2.91E+00	3.71E-01	-5.68E-01
lnlongchain	-1.57E+02	1.54E+02	-3.59E+01
AllOther/LongChain	-3.15E-01	1.14E+01	-4.45E+00
AllOther	-1.50E+01	9.71E+00	-1.65E+00
Size Parameter	1.04E+03	-1.06E+03	2.47E+02
Cation/Anion	3.13E+01	-3.65E+01	6.86E+00
lnMWCation	-4.16E+02	5.21E+02	-1.34E+02
(SO2)2N	-7.10E+02	7.72E+02	-1.86E+02
lnMWAnion	-1.23E+02	2.62E+02	-8.81E+01
intercept	-6.99E+10		

Sample Calculation

Component name = 1-methyl-3-propylimidazolium bromide

Temperature = 53.39 K

$$C_P = R[A + B(\frac{T}{100}) + C\left(\frac{T}{100}\right)^2]$$

Where $A = \Sigma(a_i \times n_i)$, $B = \Sigma(b_i \times n_i)$, $C = \Sigma(c_i \times n_i)$ and a_i, b_i and c_i are the contribution parameters of the group, i, R is the universal gas constant which is equal to 8.314472 J/mol.K, and n_i is the frequency of occurrence of the group, i.

Table S4. Group contribution parameters and frequency of occurrence of the groups for the 1-methyl-3-propylimidazolium bromide used for sample calculation using developed GCM.

Groups	ai	bi	ci	ni
Imidazolium	-1.22E+03	8.34E+02	-1.47E+02	1
Pyridinium	2.61E+03	-1.72E+03	2.90E+02	0

Pyrrolidinium	-3.22E+03	2.10E+03	-3.49E+02	0
Piperidinium	1.01E-03	-2.50E-06	0.00E+00	0
Ammonium	7.40E+00	2.64E+01	-1.87E+01	0
Phosphonium	-3.12E+02	3.74E+02	-9.80E+01	0
Thiophenium	-2.86E-01	-8.43E-01	-2.51E+00	0
quinolinium	4.77E+02	-3.15E+02	5.38E+01	0
Azepanium	4.20E-04	0.00E+00	0.00E+00	0
–H	1.19E+03	-7.75E+02	1.29E+02	3
–CH3	2.18E+03	-1.42E+03	2.23E+02	2
–CH2–	-1.13E+02	1.36E+02	-3.35E+01	2
–CH〈	2.98E-03	-2.79E-09	0.00E+00	0
–OH	2.95E+02	-7.86E+01	-2.16E+01	0
–O–	-5.46E-05	0.00E+00	0.00E+00	0
〉C〈	1.47E-04	0.00E+00	0.00E+00	0
–COO	-4.07E-05	0.00E+00	0.00E+00	0
–CN	-9.01E-01	-2.69E+00	-8.00E+00	0
–NH2	5.74E-05	0.00E+00	0.00E+00	0
–SO2–	3.28E-05	0.00E+00	0.00E+00	0
Phenyl	4.14E-06	0.00E+00	0.00E+00	0
–Benzyl	-1.63E+03	1.23E+03	-2.43E+02	0
–Vinyl	2.89E-06	0.00E+00	0.00E+00	0
–CF2–	8.73E-05	0.00E+00	0.00E+00	0
–CF3	-7.61E-05	0.00E+00	0.00E+00	0
–CH3.1	-6.15E+02	7.32E+02	-1.87E+02	0
–CH2–.1	-2.91E-05	0.00E+00	0.00E+00	0
〉CH–	-9.95E-06	0.00E+00	0.00E+00	0
〉C〈.1	-5.36E-07	0.00E+00	0.00E+00	0
〉CO	-9.30E-06	0.00E+00	0.00E+00	0
–COO–	6.12E+01	-1.39E+02	4.17E+01	0
–HCOO	8.05E-05	0.00E+00	0.00E+00	0
–OH.1	8.04E-05	0.00E+00	0.00E+00	0
–O– [–O]	8.58E+01	-1.75E+02	5.24E+01	0
–CN.1	-6.24E-05	0.00E+00	0.00E+00	0
–N– [〉N–]	-2.44E-05	0.00E+00	0.00E+00	0
–NO2	-4.71E+02	4.50E+02	-1.07E+02	0
–S–	-5.64E-05	0.00E+00	0.00E+00	0
–SO2–.1	-3.33E+02	3.63E+02	-8.90E+01	0
–CF3.1	-7.33E+02	7.44E+02	-1.77E+02	0
–CF2–.1	-5.21E+02	5.32E+02	-1.26E+02	0
–F.1	-1.51E+02	1.40E+02	-3.17E+01	0
–Cl.1	-1.38E+02	1.09E+02	-2.64E+01	0

–Br.1	-4.05E+02	3.27E+02	-7.11E+01	1
–P	-4.12E+01	6.17E+01	-1.66E+01	0
–B	1.23E+02	-1.48E+02	3.65E+01	0
–I	-7.88E+02	7.19E+02	-1.63E+02	0
CH– (ring)	-6.39E+01	4.65E+01	-8.68E+00	0
C– (ring)	-3.19E+01	2.32E+01	-4.34E+00	0
〉CO (ring)	-1.11E-05	0.00E+00	0.00E+00	0
–O– (ring)	-2.29E-06	0.00E+00	0.00E+00	0
MW	2.27E+07	-2.05E+00	1.11E+00	205.1
NormalizedMW	-3.32E+09	-1.38E+03	2.67E+02	-1.128061
Total C in Core	-1.10E+03	7.37E+02	-1.27E+02	3
Total Hetero in Core	2.81E+03	-1.84E+03	3.11E+02	2
TotalCationCores	-1.66E+03	1.30E+03	-2.71E+02	1
Nitro	-7.64E-03	2.78E-05	0.00E+00	0
Number of Rings	-2.50E+03	1.81E+03	-3.44E+02	1
Ring CH (Non-Core)	1.91E+03	-1.26E+03	2.15E+02	0
Longest Chain	-4.74E+01	-4.15E+00	7.28E+00	3
NumberofChains	-7.89E+02	5.27E+02	-7.50E+01	2
LongChain	2.91E+00	3.71E-01	-5.68E-01	43
lnlongchain	-1.57E+02	1.54E+02	-3.59E+01	3.7612
AllOther/LongChain	-3.15E-01	1.14E+01	-4.45E+00	0.418605
AllOther	-1.50E+01	9.71E+00	-1.65E+00	18
Size Parameter	1.04E+03	-1.06E+03	2.47E+02	0.343725
Cation/Anion	3.13E+01	-3.65E+01	6.86E+00	1.56375
lnMWCation	-4.16E+02	5.21E+02	-1.34E+02	-0.393552
(SO2)2N	-7.10E+02	7.72E+02	-1.86E+02	0
lnMWAnion	-1.23E+02	2.62E+02	-8.81E+01	-0.83613

All below calculations were done using excel:

A= 8.410E+09 K^{-1}, **B**= 5.22 K^{-1}, **C**= 0.809 K^{-2}, **R**= 8.314 $J \cdot mol^{-1} \cdot K^{-1}$, **T**= 53.39 K

Intercept= -6.99E+10 $J \cdot mol^{-1} \cdot K^{-1}$

$$C_P = 8.314 * [8.410E + 09 + (5.22)(\frac{53.39}{100}) + (0.809)\left(\frac{53.39}{100}\right)^2] + \text{-6.99E+10}]$$

$C_{P\,cal}$ =76.8 $J \cdot mol^{-1} \cdot K^{-1}$ (calculated from equation)

$C_{P\,pred}$ = 76.81 $J \cdot mol^{-1} \cdot K^{-1}$ (predicted by python/modelling)

$C_{P\,actual}$ = 66.92 $J \cdot mol^{-1} \cdot K^{-1}$ (experimental value)
